# DNA methylation changes underlie the long-term association between periodontitis and atherosclerotic cardiovascular disease

**DOI:** 10.3389/fcvm.2023.1164499

**Published:** 2023-04-21

**Authors:** Mohamed Omar, Maria Alexiou, Umar R. Rekhi, Konrad Lehmann, Aneesh Bhardwaj, Cole Delyea, Shokrollah Elahi, Maria Febbraio

**Affiliations:** School of Dentistry, College of Life Sciences, University of Alberta, Edmonton, AB, Canada

**Keywords:** periodontal disease, atherosclerosis, epigenetics, DNA methylation, *Porphyromonas gingivalis*

## Abstract

Periodontitis, the leading cause of adult tooth loss, has been identified as an independent risk factor for cardiovascular disease (CVD). Studies suggest that periodontitis, like other CVD risk factors, shows the persistence of increased CVD risk even after mitigation. We hypothesized that periodontitis induces epigenetic changes in hematopoietic stem cells in the bone marrow (BM), and such changes persist after the clinical elimination of the disease and underlie the increased CVD risk. We used a BM transplant approach to simulate the clinical elimination of periodontitis and the persistence of the hypothesized epigenetic reprogramming. Using the low-density lipoprotein receptor knockout (*LDLR^o^*) atherosclerosis mouse model, BM donor mice were fed a high-fat diet to induce atherosclerosis and orally inoculated with *Porphyromonas gingivalis* (*Pg*), a keystone periodontal pathogen; the second group was sham-inoculated. Naïve *LDLR*^o^ mice were irradiated and transplanted with BM from one of the two donor groups. Recipients of BM from *Pg*-inoculated donors developed significantly more atherosclerosis, accompanied by cytokine/chemokines that suggested BM progenitor cell mobilization and were associated with atherosclerosis and/or PD. Using whole-genome bisulfite sequencing, 375 differentially methylated regions (DMRs) and global hypomethylation in recipients of BM from *Pg*-inoculated donors were observed. Some DMRs pointed to the involvement of enzymes with major roles in DNA methylation and demethylation. In validation assays, we found a significant increase in the activity of ten-eleven translocase-2 and a decrease in the activity of DNA methyltransferases. Plasma S-adenosylhomocysteine levels were significantly higher, and the S-adenosylmethionine to S-adenosylhomocysteine ratio was decreased, both of which have been associated with CVD. These changes may be related to increased oxidative stress as a result of *Pg* infection. These data suggest a novel and paradigm-shifting mechanism in the long-term association between periodontitis and atherosclerotic CVD.

## Introduction

In most developed countries, cardiovascular disease (CVD) remains the leading cause of death, despite major advances in prevention and treatment options. In Canada, atherosclerosis, which underlies CVD, accounts for 20% of all deaths (second leading cause), which translates into a death every 7 min (1). The evidence for chronic periodontal disease (PD) as an independent risk factor for CVD has increased significantly in recent years, with studies on both human and mouse models suggesting a role (2–7). In total, 20%–24% of Canadians aged 20–79 years have moderate to severe PD, while 47% of the US population >30 years has periodontitis; the rate is 64% for adults 65 years and older (1, 8). While the underlying mechanism for the association between PD and CVD remains undetermined, evidence supports increased systemic inflammation and oxidative stress, endotoxemia, and endothelial dysfunction, among others (9).

*Porphyromonas gingivalis* (*Pg*), a Gram-negative anaerobe, is a keystone pathogen in chronic adult PD (10–12). This refers to its ability to control the mass/composition of the oral plaque community. *Pg* elicits a powerful host immune response that leads to gum disengagement from teeth, pockets of inflammatory cells, and bone destruction (13). Our previous work showed that oral inoculation with *Pg* in an atherosclerosis-prone mouse model, the low-density lipoprotein receptor knockout (*LDLR^o^*), significantly increased atherosclerosis aortic lesion burden (4). We found increased systemic inflammation related to toll-like receptor 2 signaling and interleukin (IL)1beta generation, a key cytokine in PD and atherosclerosis (4, 14–16). Our findings were consistent with human studies that show that the effects of *Pg* are not limited to the periodontium and add supportive evidence for PD as a risk factor for inflammatory diseases other than atherosclerosis, such as diabetes, chronic kidney disease, and obesity (9).

Epidemiological and experimental human studies show that atherosclerotic PD patients are at higher risk of heart attack and stroke (17, 18). Successful treatment of PD has been shown to have no effect or short-term changes on markers of inflammation (19–22). The impact of therapy on CVD progression, events, or mortality remains unclear (19, 20, 23, 24). In one study, patients who had all teeth extracted and thus verifiable eradication of PD were followed up for up to 17 years and did not show lower CVD risk (23). Despite successful PD treatment, the potential for sustained higher risk of CVD suggests that PD may cause long-term changes and led to the hypothesis that these changes affect immune cells and their precursors, leading to an increased proatherogenic state, despite eradication of the pathogen.

“Metabolic memory” is the concept that hyperglycemia can induce persistent, long-term effects in cells after subsequent normoglycemia is achieved (25). Thus, if tight glucose control is not initiated immediately, despite such therapy, people with diabetes remain at higher risk for CVD due to permanent cellular changes (26, 27). Studies implicated epigenetic changes in inflammatory genes and increased reactive oxygen species (ROS) production as a driving mechanism (25, 28–32). Epigenetic changes have also been shown due to PD, aging, diet, atherosclerosis, and infection (33–40). Epigenetic modifications alter gene expression in the absence of DNA sequence change and include DNA methylation, histone modifications, and changes in the expression of noncoding RNAs (41). Transgenerational epigenetic effects of diet have been shown in patients/mice and illustrate the potential for environmental factors/CVD risk factors to induce such changes in stem cells (42–47).

Atherosclerotic lesion macrophages derive from proliferation at the site and through constant replenishment from the BM (48, 49). Thus, atherosclerotic risk factors may achieve long-lived effects *via* epigenetic alterations in macrophage precursors. Without downplaying the roles of other immune and vascular cells in lesion development, or the potential impact of direct *Pg* infection of lesions, this study focuses on the epigenomic effects of *Pg* in the development of macrophage phenotype. Our data show that *LDLR^o^* mice transplanted with BM from *LDLR^o^* mice orally inoculated with *Pg* had greater atherosclerosis than mice transplanted with sham-treated mouse BM. This was accompanied by differential gene expression in macrophages and genomic changes in methylation patterns 5 months *post-transplantation*, all in the absence of the pathogen.

## Results

### Oral lavage with *Pg* elicited an increased inflammatory response in macrophages from donor mice

**Figure 1:**
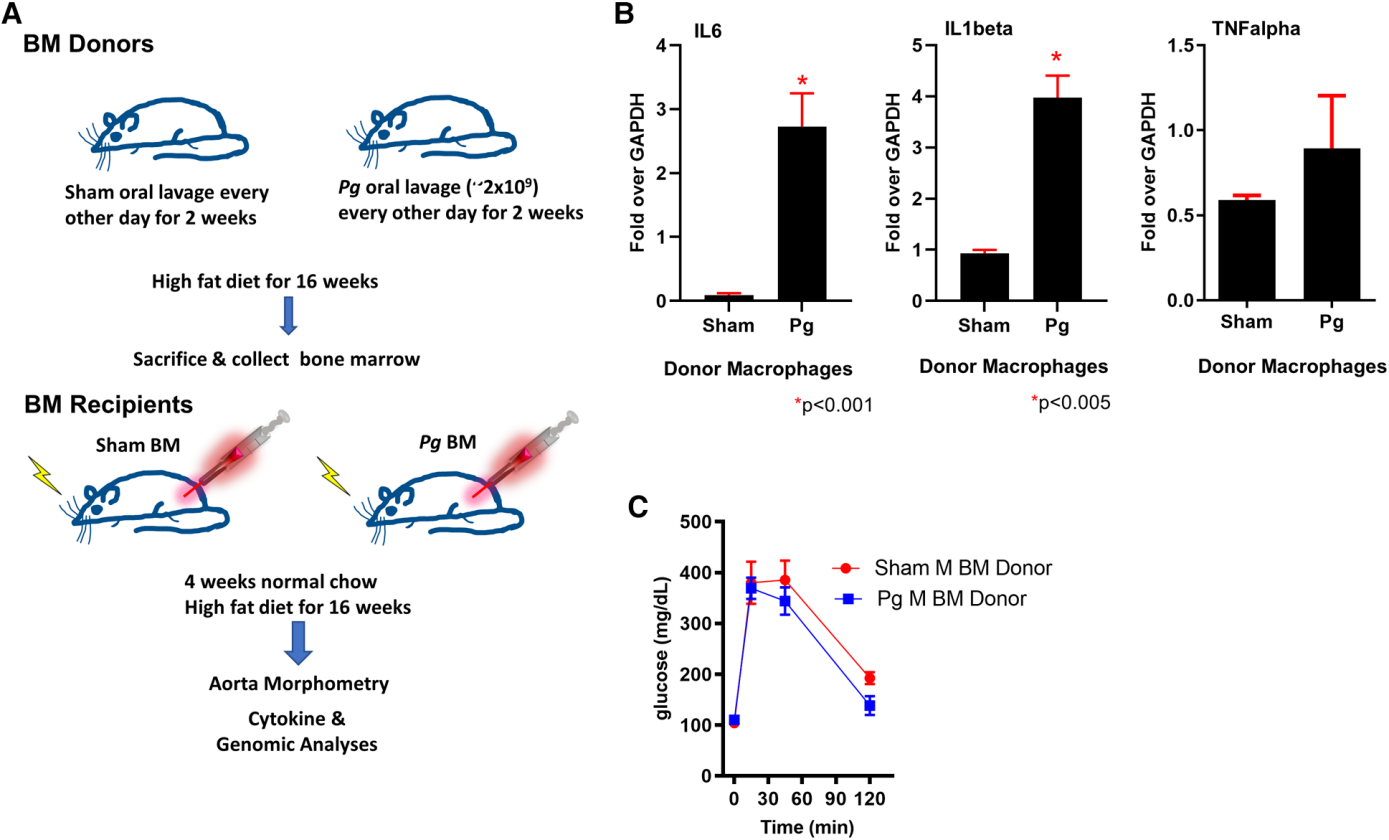
(**A**) Study design. Male and female donor BM *LDLR^o^* mice were inoculated by oral lavage with a solution containing *Pg* or vehicle (sham) every other day for 2 weeks, coincident with high-fat diet feeding. After 16 weeks, femurs were removed for isolation of BM. The recipient *LDLR^o^* mice (*n* = 9–15/group) were lethally irradiated and transplanted with gender-matched BM from the donor groups. The mice were fed normal chow for 4 weeks and then a high-fat diet for 16 weeks. (**B**) qPCR analysis of macrophages isolated from donor mice showed significantly increased expression of IL6 and IL1beta; TNFalpha was no different. These cytokines are associated with a chronic PD infection. **(C)** Glucose tolerance testing of male BM donor mice (*n* = 4 sham, *n* = 5 Pg).

The most commonly isolated human *Pg* strain (33277) was used to infect *LDLR^o^* mice of both sexes by oral lavage (3, 4, 50); control mice were sham-inoculated with vehicle alone (see Figure 1A for study design). Mice were fed a high-fat diet (21% fat, 0.15% cholesterol) to promote atherosclerosis, which continued for 16 weeks. We designed this experiment to mimic a patient with CVD risk factors (hyperlipidemia) with and without PD. To determine the success in creating a chronic inflammatory condition in mice after oral lavage with *Pg*, we investigated the expression of cytokines known to be significantly associated with PD (14, 15). qPCR of macrophage lysates isolated 16 weeks after initial inoculation with *Pg* showed a significant 3- to 4-fold increase in IL1beta and IL6 compared to sham-inoculated mice; tumor necrosis factor (TNF) alpha was not significantly elevated (Figure 1B). Donor mice were of similar weight (data not shown) and had similar insulin sensitivity as gauged by glucose tolerance testing (Figure 1C).

### Male mice transplanted with BM from *Pg*-inoculated donors had greater atherosclerosis lesion burden than male mice transplanted with BM from sham-inoculated mice

*LDLR^o^* mice of both sexes were irradiated (lethal dose) following a standard protocol. Because we transplanted *LDLR^o^* BM into *LDLR^o^* mice, it was impossible to directly confirm engraftment success. Accordingly, a control experiment was performed, whereby *CD36^o^* mice were transplanted with wild-type BM. Chimerism was assessed by polymerase chain reaction (PCR) using isolated DNA from white blood cells from recipient mice, collected 4 weeks after irradiation. All recipient mice showed only the *CD36* wild-type genotype under conditions where there was successful amplification of *CD36* wild-type and *CD36^o^* alleles (Supplementary Material Figure S1A). In a second experiment, we transplanted *CD36* wild-type mice with *CD36^o^* BM. White blood cells were isolated and subjected to flow cytometry analysis. Monocytes were selected by gating on the CD11b- and CD11c-positive population, and then the expression of CD36 was measured. Cells from nontransplanted *CD36* wild-type mice showed a CD36^+^ monocyte population of 18.6%, whereas those transplanted with *CD36^o^* BM showed less than 1% positive cells in three different recipients (Supplementary Material Figure S1B) 4 weeks after transplantation. These control experiments indicated that our irradiation and transplantation protocols were successful.

**Figure 2:**
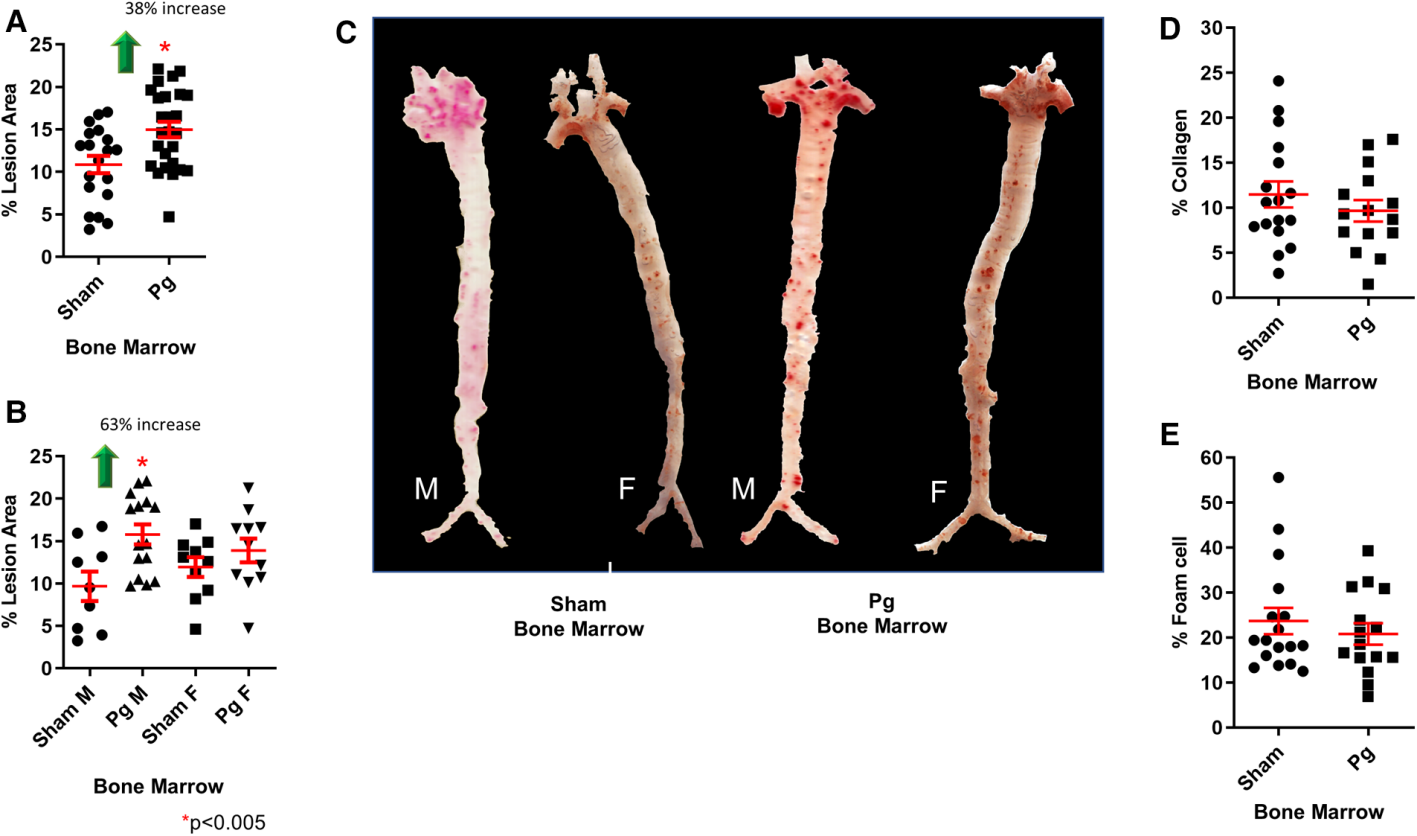
(**A**) *En face* morphometric analysis of oil red O-stained aortae showed a significant 38% increase in lesion area in mice that received BM from *Pg*-inoculated donor mice compared with those that received BM from sham-inoculated donors. (**B**) Sex-specific analysis showed a significant 63% increase in male mice that received BM from *Pg*-inoculated donor mice compared with those that received BM from sham-inoculated donors. There was no significant difference between the female groups. M = male; F = female; *n* = 9–15/group. **(C)** Oil red O-stained aortae from the groups. (**D**) Percent collagen content and (**E**) percent foam cell in similarly sized lesions from the aortic sinus (*n* = 5/group).

**Figure 3:**
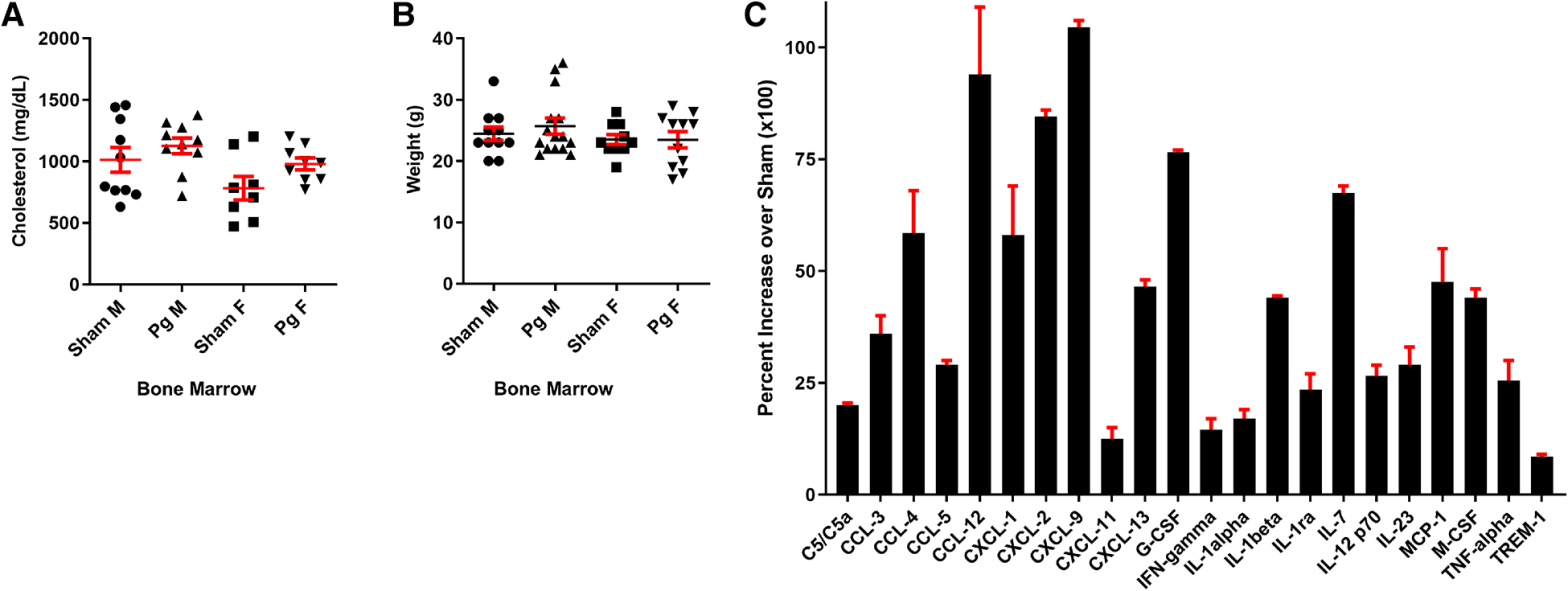
(**A**) Total plasma cholesterol and (**B**) weight at sacrifice. M = male; F = female (*n* = 8–15/group). (**C**) Significantly different cytokines/chemokines, represented as fold over sham, in macrophage lysates from male mice that received BM from *Pg*-inoculated donor mice (*n* = 5/group, pooled), out of a total of 40 measured.

As shown in Figure 2A, mice that received BM from *Pg*-inoculated mice had significantly more (38%) atherosclerotic lesions than those that received BM from sham-treated mice. Sex-based analysis showed a significant 62% increase in atherosclerotic lesions in male mice receiving *Pg* BM than in those receiving sham but no significant difference between female groups (Figure 2B). Representative aortae are shown in Figure 2C. The reason for this sex effect is unknown and requires further study. Examination of similarly sized isolated lesions in the aortic sinus for collagen and macrophage foam cell content showed no differences (Figures 2D,E). Fasting plasma cholesterol levels showed no significant differences between the groups (Figure 3A). Because obesity is a risk factor for atherosclerosis and also impacts inflammation and insulin resistance, we compared mouse weight at sacrifice. Again, there were no significant differences between the groups (Figure 3B). This also implied that inoculation with *Pg* had no effect on mouse food intake. These data indicated that well-established risk factors for atherosclerosis did not have a role/confound our results.

### Male mice transplanted with BM from *Pg-*inoculated donors had a macrophage cytokine profile that suggested BM mobilization and response to PD pathogens

In circulating macrophages, the cytokines shown in Figure 3C were statistically different between male mice receiving *Pg* BM and those receiving sham (51). Several of these, including granulocyte colony-stimulating factor (G-CSF) and IL1alpha, induce expansion and mobilization of immune cells from the BM (52–54). Macrophage colony-stimulating factor (M-CSF) induces cells of the myeloid lineage to differentiate into monocytes, macrophages, and dendritic cells (55). IL23, G-CSF, M-CSF, TNFalpha, chemokine c-c motif ligand (CCL)3, CCL5, complement component (C) 5/5a, c-x-c motif chemokine ligand (CXCL)9, and CXCL13 have been shown to induce osteoclastogenic differentiation and/or bone loss (15, 52, 55–62). IL23 is considered to have a major role in the pathogenesis of PD (56). Thus, the cytokine profile was consistent with response to a PD infection in these mice, 20 weeks post-transplant, in the absence of the pathogen.

### Whole-genome bisulfite sequencing (WGBS) showed a differentially methylated profile in macrophages from male mice that received BM from *Pg*-inoculated donors

**Figure 4:**
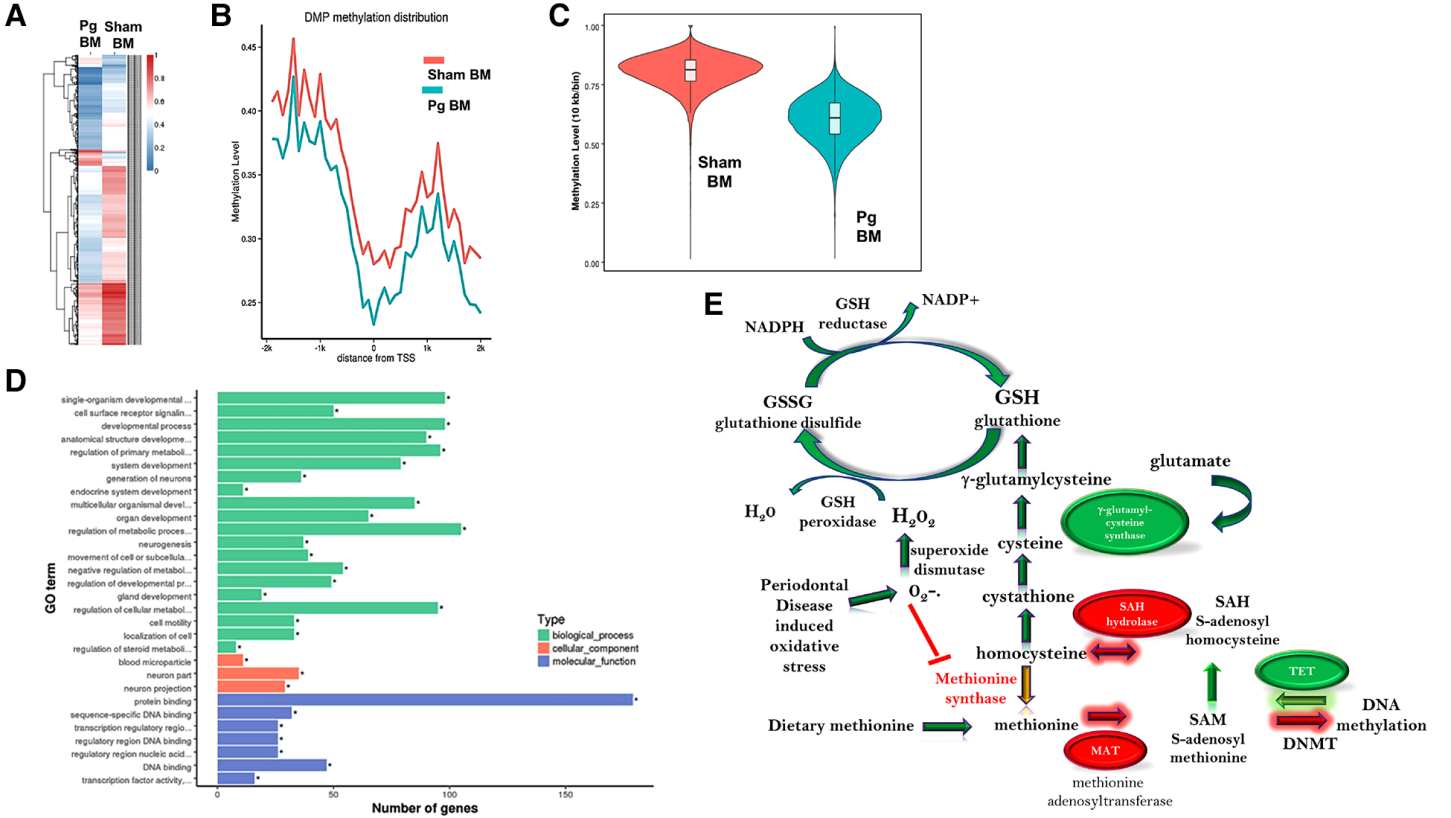
(**A**) Hierarchal cluster analysis of each differentially methylated promoter (DMP) presenting methylation values of 0 (deep blue, less methylation) to 1 (deep red, more methylation), where 0 is unmethylated and 1 is completely methylated. Each line represents one DMP, and the columns represent the different groups. Male mice that received BM from *Pg*-inoculated donors showed more intense blue, representing less methylation, than male mice that received BM from sham-inoculated donors. (**B**) Promoter methylation at the single nucleotide level 2 kb upstream and downstream from the transcription start site (TSS). Male mice that received BM from *Pg*-inoculated donors showed less methylation throughout this region. **(C)** Violin plot depicting the global DNA methylation levels of male mice that received BM from *Pg*-inoculated and sham-inoculated donors. Using 10-kb bins, the methylation level was calculated based on the ratio of methylated cytosines to total (unmethylated + methylated cytosines). The width of each violin represents the number of methylated sites at the corresponding methylation level. The boxes represent the 25% and 75% interquartile ranges, and the horizontal line represents the median level of methylation. Male mice that received BM from *Pg*-inoculated donors showed lower levels of methylation overall. (**D**) Gene ontology terms and the number of genes in each group that were found to be differentially methylated in male mice that received BM from *Pg*-inoculated donors compared to those that received BM from sham-inoculated donors. (**E**) Overall schematic of the intersection between DNA methylation/demethylation pathways, the transsulfuration pathway, and reactive oxygen species detoxification. The circles represent genes that were found to be hypo methylated (green) or hyper methylated (red) in our WGBS results. The arrows represent whether a path is predicted to be favored (green) or reduced (red, yellow) based on our results. The predicted intersection of oxidative stress from chronic PD with these pathways is also shown.

We pooled (5/group) and isolated DNA from macrophages obtained at sacrifice from male mice and performed WGBS to determine whether methylation differences could account for the observed changes in the phenotype. The bisulfite conversion rate was greater than 99.6%. Bismark software was used to align bisulfite-treated reads to the mouse reference genome (63). The unique mapping rate was 74.2–79.7%, and the duplication rate was 13.3–17.2%. Mean coverage was between 15.99× (Sham BM) and 19.05× (*Pg* BM). In total, 90% of sequences in both samples had a coverage depth of 5×, and at 10×, the values were 73.95% (sham BM) and 83.48% (*Pg* BM). A recent study compared differentially methylated regions (DMRs) identified at different sequence depths and concluded that a sequence depth of 5× to 15× is sufficient and that higher depths are not justified (64). Cluster analysis of differentially methylated promoters is shown in Figure 4A. Promoter methylation changes occurred at the region upstream of the transcription start site (Figure 4B); this localization further suggested the potential for change in gene expression. DMRs were assessed by a sliding window approach (65), which incorporates statistical methods (Fisher's exact test) and repeated measures across the genome and is appropriate to compare samples that do not have replicates. A false discovery rate was obtained and used to adjust *p*-values. DMRs were defined as having at least 2-fold change, at least 10 cytosines, a methylation difference of at least 0.2, and adjusted *p* < 0.05. Comparing samples *Pg* BM recipients vs. sham BM recipients, there were 158 hypermethylated regions and 217 hypomethylated regions, for a total of 375 DMRs that reached significance after *p*-value adjustment (Supplementary Material Figures S1, S2). Many DMRs were in cytosine–guanine (CpG) island shelf regions. As shown in Figure 4C, DNA from macrophages from male mice that received BM from *Pg-*inoculated donors had an overall decrease in genomic methylation levels compared with DNA from macrophages from male mice that received BM from sham-treated mice. In general, hypomethylation favors increased gene expression. Table 1 summarizes significantly differentially methylated genes related to specific processes.

**Table 1:** DMRs that were statistically different in mice that received BM from *Pg*-inoculated donors relative to sham-treated.

FA Ox, glycol	Glutamine methionine	M1 vs. M2	Athero	Athero	Epigen	Bone, osteoclast	NFkB
Vps54	Gclm	Grb10 (m2)	Lrp8	Galnt2	Rbm15b	LRP8	Sirt1
Ppp2r5a	Grm4	Gclm (mox)	Igf2r	Apoa1	Gse1	Igf2r	Tet2
Nrtn	Mat1a	Hoxa3 (m2)	Zfpm1	Apob	Tet2	Mn1	Plxna1
Foxk2	Ahcy	Manf (m2)	Gclm	Apoe	Gon4l	Fam19a5	Atp11a
Acot4	Slc17a7	Ddx4 (m2)	Manf	Jund	Setd8	Nrf2f2	Spat2l
Pde3b		Adcy2 (m2)	Grb10	Ahcy	Bcor	Pmepa1	Grsf1
Galnt2		Igf2r (m2)	Sh2b3	Cldn5	Sirt1	Fignl1	Lmx1b
Dvl1		Mgat3 (m2)	Igf2r	Dmd	Kmt2b	Sh3pxd2b	Gfi1
Igf2r		Casz1 (m1)	Lsamp	Pabpc1	Setd8	Wt1os	Otud7a
Egln1		Setd8 (m1)	Wt1	Foxa2		Zfp521	Tnfaip2
Scd1		Sirt1 (m2)	Slc23a2	Hsp8		Hivep3	Prkce
Mat1a		Rbpj (m2)	Furin	Ikbkg		Pou4f1	Pde3b
Aldob		Aldob (m1)	Adcy2	Cebpa		Adcy2	Ikbkg
Apoa1			Tnfaip2	Actb		Sirt1	Cebpa
Atp5d			Bcor			Foxo6	Usp7
Cebpa			Hspa1b			Egln1	Pigr
Car3			Fkbp5			Myo10	
Foxa2			Prkce			Dvl1	
			Egln1			Rbpj	
			Rbpj			Fkbp5	
			Timp1			Cebpa	
			Ptprn2			Slc17a7	

Green, hypomethylated; red, hypermethylated; FA Ox, fatty acid oxidation; Glycol, glycolysis; Athero, atherosclerosis; Epigen, epigenetics.

Gene ontology analysis significance was determined after *p*-value correction (Supplementary Material Figures S3, S4). The top biological processes uncovered were related to cellular metabolism and the top molecular function was related to protein binding (Figure 4D). Using the Kyoto Encyclopedia of Genes and Genomes (KEGG), the top pathways hypermethylated were peroxisome proliferator-activated receptor signaling, cysteine and methionine metabolism, and glycolysis/gluconeogenesis (Supplementary Material Figure S1). Glutamine/glutamate was the top hypomethylated pathway (Supplementary Material Figure S2). In macrophages, the intersection between glutamine/glutamate, the tricarboxcylic acid cycle, and cysteine/methionine and glutathione pathways have recently emerged as key drivers of metabolism and macrophage polarity (66–70).

### Hypermethylation of the rate-limiting enzymes in the methionine cycle in male mice that received BM from *Pg*-inoculated donors

**Figure 5:**
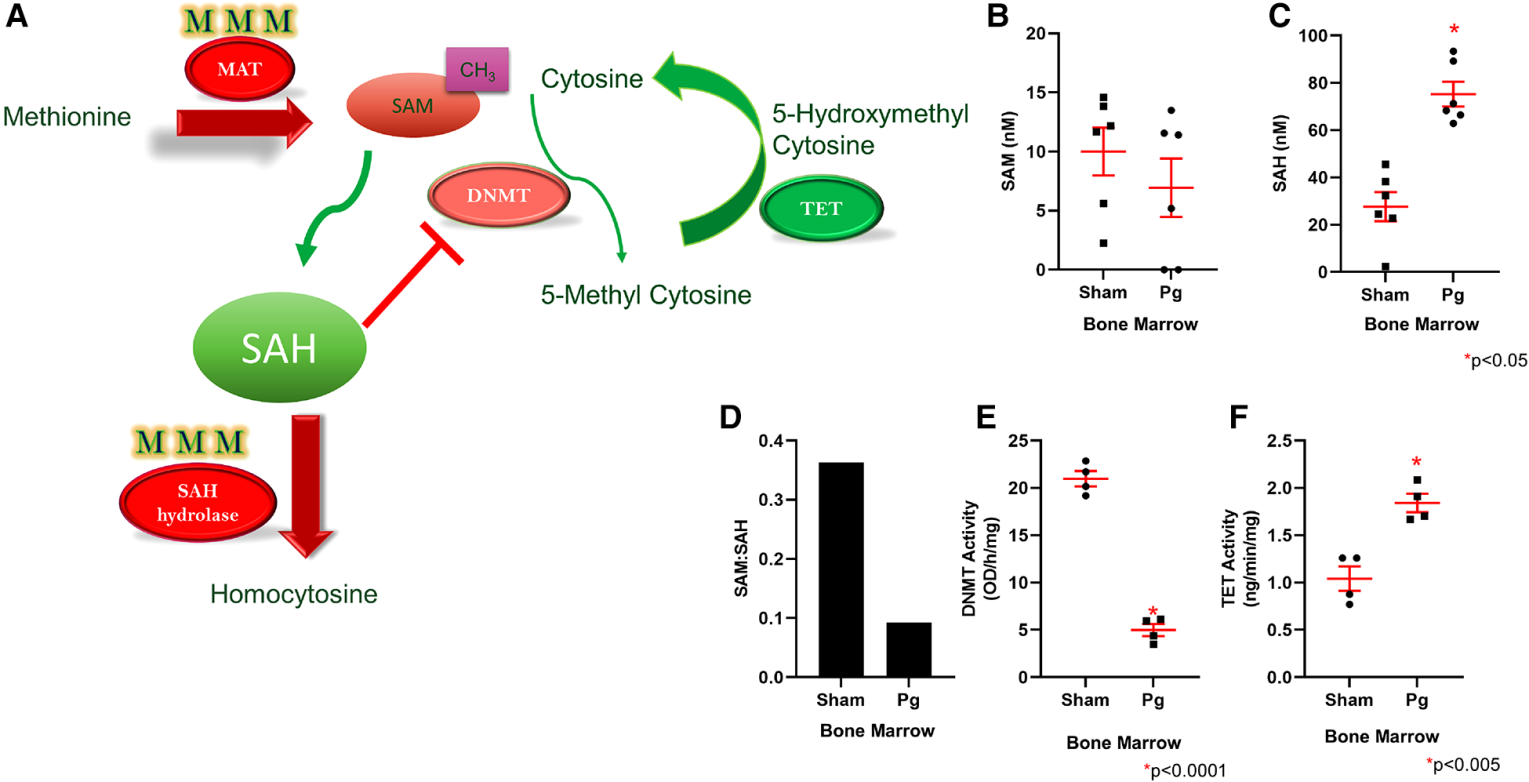
(**A**) Methionine is converted to SAM through the action of MAT, which was found to be hypermethylated in mice that received *Pg*-BM; SAM levels are therefore predicted to be lower, and this may lead to lower DNMT activity. SAH hydrolase, which converts SAH to homocysteine, was also hypermethylated in mice that received *Pg*-BM. SAM conversion to SAH was predicted to be unaffected, but SAH conversion to homocysteine was predicted to be inhibited, leading to increased levels of SAH. Increased SAH inhibits DNMT activity. TET was found to be hypomethylated in mice that received *Pg*-BM; therefore, TET activity was predicted to be higher. Plasma levels of SAM (**B**) and SAH (**C**) and the ratio of SAM:SAH (**D**) in male mice transplanted with BM from *Pg-* or sham-inoculated donors (*n* = 6/group). Levels of SAM did not differ, but mice that received *Pg* BM had significantly higher SAH levels. The ratio of SAM:SAH was much lower in these mice. Nuclear extracts were isolated from male mice transplanted with BM from *Pg-* or sham-inoculated donors, and the activities of DNMT (**E**) and TET (**F**) were measured. Mice that received *Pg* BM had significantly lower DNMT activity and significantly higher TET activity (*n* = 4/group).

Homocysteine, a sulfur-containing amino acid, is critically positioned at the intersection between the methionine cycle and the transsulfuration pathway (71). It is produced from methionine in the methionine cycle and can then recycle back to methionine or be converted to cysteine in the transsulfuration pathway to generate glutathione (Figure 4E). In the methionine cycle, the methyl group of methionine is activated by addition of adenosine to sulfur from adenosine triphosphate, a process catalyzed by methionine adenosyl transferase (MAT). This converts methionine to S-adenosylmethionine (SAM), the universal methyl donor (71). SAM is used by methyltransferases, including DNA methyltransferases (DNMT), and in the process, SAM is converted to S-adenosylhomocysteine (SAH) (Figure 5A). WGBS data showed significant hypermethylation of *MAT* in male BM recipients from *Pg*-inoculated donors compared to sham-inoculated donors. Importantly, this hypermethylation was in the 3′ untranslated region. Methylation of this specific region has been associated with degradation of MAT and, in turn, depletion of SAM. These data suggested that hypermethylation of *MAT* and the subsequent attenuation of the methionine cycle could at least be partially responsible for the hypomethylation observed.

SAH, a potent negative feedback inhibitor of methylation, is hydrolyzed to homocysteine and adenosine by SAH hydrolase (SAHH) in a reversible process (71, 72). SAHH is the only enzyme that catalyzes this reaction and hence is critical (73). In fact, deletion of *SAHH* in mice results in embryonic lethality, and deficiency of SAHH in humans is associated with Wilson's disease, an incurable metabolic disorder (73). WGBS data identified a hypermethylated DMR corresponding to *SAHH* in male BM recipients from *Pg*-inoculated donors compared to sham-inoculated donors. This increased methylation was located in CpG islands and CpG shores, both of which have been strongly associated with decreased gene expression. Therefore, the hypermethylation of *SAHH* strongly suggested a decreased expression of SAHH. Being the only enzyme capable of the reversible hydrolysis of SAH to homocysteine (73), this suggested that these mice would experience an increase in SAH or homocysteine or both (Figure 5A). Several studies have identified a strong, significant, and possibly synergistic association between increased levels of homocysteine and/or increased levels of SAH and atherosclerotic CVD (74, 75).

Given the critical opposing roles of SAM and SAH in the methionine cycle, we measured and compared the plasma levels of SAM and SAH in male BM recipients to validate the WGBS data with regard to hypermethylation of *MAT* and *SAHH* genes. We found comparable plasma SAM levels in both groups (Figure 5B). In contrast, SAH levels were ∼3× higher in BM recipients from *Pg*-inoculated donors than sham-inoculated donors (Figure 5C). The ratio of SAM:SAH is a measure of cellular methylation capacity. As shown in Figure 5D, mice that received BM from *Pg*-inoculated donors had a much lower SAM:SAH than those that received BM from sham-inoculated mice, which further supported our finding of DNA hypomethylation in these mice. These data were from a separate cohort of transplanted mice, obtained ∼11 months post-transplantation, suggesting robust, replicable epigenetic changes resulting from *Pg* infection in the context of an atherosclerotic mouse model.

### Recipients of *Pg*-inoculated donor BM had significantly lower DNMT activity and higher TET activity than recipients of sham-inoculated donor BM

Given that increased SAH has been associated with genomic global hypomethylation (73, 74) and significant inhibition of SAM-dependent methyltransferases (76), we next compared the activity of DNMT in nuclear extracts isolated from BM-derived macrophages (BMDM) from the two groups of mice. Our analysis showed that DNMT activity in BM recipients of sham-inoculated mice was 4× higher than that in BM recipients of *Pg*-inoculated mice (Figure 5E), an inhibition of more than 80% in the latter. These data suggested that differences in DNMT activity, and not gene expression, could account for hypomethylation of genes observed in BM recipients of *Pg*-inoculated mice.

DNA methylation is governed by dual dynamic processes: methylation by DNMTs and demethylation by the ten-eleven translocation (TET) family of 5-methylcytosine dioxygenases. Sequential oxidation of 5-methylcytosine to 5-hydroxymethylcytosine (5hmC), 5-formylcytosine (5fC), and 5-carboxycytosine (5caC) is catalyzed by TET enzymes (77, 78). Given the significant inhibition of DNMT activity, we next investigated the demethylation process by similarly analyzing and comparing TET activity in nuclear extracts from BMDM from the two groups of mice. Our analysis showed that there was ∼1.7× higher TET activity in mice that received BM from *Pg*-inoculated donors compared to mice that received BM from sham-inoculated donors (Figure 5F). This suggested that TET-driven demethylation may contribute to the observed hypomethylation of genes in mice that received BM from *Pg*-inoculated donors. In line with this, the WGBS data showed significant hypomethylation of DMRs corresponding to *TET2* in those mice. The location of this decreased methylation included CpG islands, shores, and promoter regions. Hence, our WGBS data strongly suggested that TET2 was overexpressed in these mice.

### Differences in apolipoprotein (apo) expression may contribute to increased oxidative stress and atherosclerosis in *Pg* BM recipients

**Figure 6:**
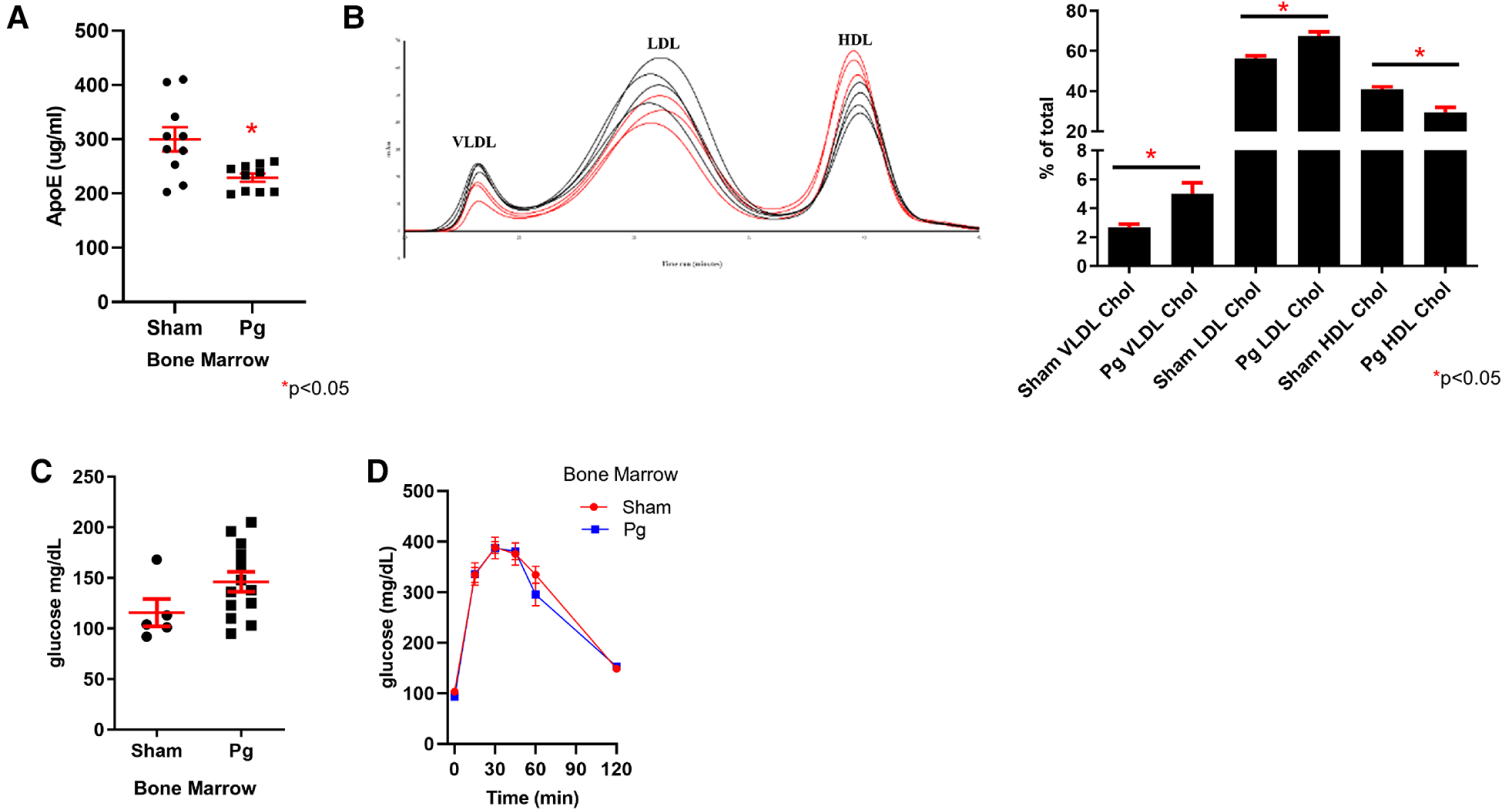
(**A**) ELISA for apoE in plasma from male mouse recipients of sham or *Pg* BM (*n* = 10/group). (**B**) Chromatographs of FPLC separation of lipoproteins and cholesterol analysis from male mouse recipients of sham or *Pg* BM (*n* = 3 sham; *n* = 4 *Pg*). Quantification of peaks as the percent of the total is shown adjacent. (**C**) Fasting glucose and (**D**) glucose tolerance testing of male mouse recipients of sham or *Pg* BM (*n* = 4 sham; *n* = 5 *Pg*).

*ApoE* was hypermethylated in the WGBS dataset of BM recipients of *Pg*-inoculated donors. Using a mouse-specific ELISA, we validated this predicted decrease in apolipoprotein E (apoE) expression (Figure 6A). Lipoprotein profiling data showed increased very low density lipoprotein (VLDL) and low density lipoprotein (LDL) cholesterol, which would be affected by reduced expression of apoE (79). In addition to its role in lipoprotein metabolism, apoE protects against oxidation of proteins, including LDL. WGBS data also showed hypermethylation of *apoA1.* Decreased apoA1 would be predicted to lead to decreased HDL, consistent with the lipoprotein profile analysis. There were no differences in triacylglycerol among the fractions (Supplementary Material Figure S2). Fasting glucose and glucose tolerance testing revealed no differences between the groups (Figures 6B,C), suggesting that insulin sensitivity was similar.

## Discussion

The pathophysiology of atherosclerosis is multifactorial, wherein the interplay among multiple risk factors increases the potential for initiation or worsening of the disease. PD, a pathogen-driven chronic inflammatory disease, is the most common oral disease and the leading cause of tooth loss in adults (80). Although the mechanisms have not been completely elucidated, in the last few decades, studies have associated PD with atherosclerosis, and in 2012, the American Heart Association identified PD as an independent risk factor for atherosclerotic vascular diseases (2, 81–83). There are also data to suggest that treatment of PD, and even eradication through removal of all teeth, does not eliminate the subsequent risk of cardiovascular events (19, 20, 23). This is similar to other CVD risk factors, including smoking and diabetes. In this study, we sought to probe this long-term sustained effect and the potential underlying mechanism.

BM transplant reconstitutes hematopoietic cells and the immune system of the lethally irradiated recipient mouse with that of the donor. In our model, we hypothesized that this would manifest as increased atherosclerotic lesion burden in the recipients of BM from donors orally inoculated with *Pg* compared with those that received BM from sham-inoculated donors. Twenty weeks after transplantation, we found that mice that received BM from the *Pg*-inoculated donors developed 38% more atherosclerosis lesions than mice that received BM from sham-inoculated donors. Interestingly, stratification according to sex revealed that the increase was more pronounced and only significant in male mice. This difference between male and female mice is in line with studies showing sex-based differences in the immune response (84). One study showed that male mice produced significantly more IL6 and lipopolysaccharide (LPS)-binding protein upon LPS treatment compared to female mice, and *in vitro*, male peritoneal macrophages expressed higher levels of IL1beta and TLR4 upon LPS stimulation (85). It is interesting that this difference in atherosclerosis lesion burden was not observed when mice were orally inoculated directly (i.e., both males and females showed increased atherosclerosis compared to sham-inoculated controls) (4). This suggests that epigenetic changes in BM stem cells only occurred in male mice; this intriguing possibility is for future study.

WGBS is considered the gold standard for an unbiased global approach to quantitative assessment of DNA methylation of the entire genome (86). Our data indicated a global hypomethylation in recipients of BM from *Pg*-inoculated donors, in line with major studies in the field that associated DNA global hypomethylation with increased atherosclerosis in human and animal studies. One study showed a 9% decrease in DNA methylation in advanced human atherosclerotic lesions compared to that in non-diseased arteries, and a 7% decrease in DNA methylation in high-fat diet-induced atherosclerotic *ApoE^o^* mice compared to that in chow-fed *ApoE^o^* mice (87). In the same study, the authors assessed global methylation levels in rabbits after balloon denudation of the aorta. Proliferation of intimal smooth muscle cells was associated with a 15% decrease in DNA methylation level (87). This level of hypomethylation was evident in normal chow-fed and high-fat-diet-fed rabbits, suggesting that the decreased methylation was independent of cholesterol levels in this model (87). A more recent study reported a 2.5-fold decrease in global DNA methylation in human atherosclerotic arteries compared to that in healthy arteries, and this was associated with increased gene expression (88). Furthermore, a 2015 human study associated hypomethylation of long interspersed nuclear element-1 (LINE-1) with an increased risk of myocardial infarction (89). Since LINE-1 compromises ∼17% of the human genome, the extent of its methylation is considered a surrogate marker of global DNA methylation (90). The finding of DNA hypomethylation as a characteristic of atherosclerosis is not conclusive; there are also studies that suggest that global hypermethylation is a characteristic of atherosclerosis (91). Why some studies identified hypomethylation and others hypermethylation in atherosclerosis could represent population-based differences or could be due to an inherent biological issue. Our study differed from these in that we used non-lesion macrophages for profiling to understand changes in bone marrow-derived monocytes/macrophages as a result of PD prior to their residency in atherosclerotic plaque.

Our WBGS data identified 217 hypomethylated and 158 hypermethylated DMRs and hypomethylation of promotors close to the transcription start site. This localization strongly suggested an impact on gene expression (92). The analysis and validation of each of these DMRs are warranted but go beyond the scope of the current study. However, it is noteworthy that many of these genes can be categorized into pathways that affect macrophage phenotype, inflammation, metabolism, and atherosclerosis. The control group was treated the same, so the difference between the groups is the exposure of one set of BM donors to *Pg* and an increased atherosclerosis lesion burden. Interestingly, many DMRs were also associated with TLR/nuclear factor kappa B (NFkB) signaling and osteoclast biology. Thus, it appears that these cells were responding to the PD pathogen, although they had never been infected with *Pg*.

The DMRs identified showed enrichment for genes associated with active methylation or active demethylation. *MAT*, which encodes the enzyme responsible for catalyzing the conversion of methionine to the universal methyl-donor, SAM, was hypermethylated in mice that received BM from *Pg*-inoculated donors, which suggested decreased expression. *SAHH* was also hypermethylated; SAHH catalyzes hydrolysis of SAH, a potent methylation inhibitor, to homocysteine and adenosine. This is a reversible reaction, and interestingly, SAHH underexpression has been associated with overexpression of both SAH and homocysteine (73–75). In contrast, *TET2*, which encodes one of the demethylation enzymes, and *Sirt1*, the gene for an enzyme that modulates the activity of DNMT1, were hypomethylated. Considering all these “methylation-determining” enzymes being differentially methylated, we sought to investigate the potential mechanism underlying the observed global hypomethylation in mice that received BM from *Pg*-inoculated donors and its association with the increased atherosclerosis observed.

DNMT1, the maintenance methyl transferase enzyme, has been repeatedly reported to be underexpressed and inhibited in cases of global hypomethylation and has been associated with increased atherogenesis (88, 93). Our activity assay could not differentiate among DNMTs but showed that DNMT was four times more active in mice that received BM from sham-inoculated donors than in those that received BM from *Pg*-inoculated donors. This is in line with a study that investigated the effect of hyperlipidemia and hyperhomocysteinemia on atherogenesis in *ApoE^o^* mice and found that increased atherosclerosis was associated with global DNA hypomethylation, decreased DNMT1 gene expression and activity (94). Similar results have been reported in human studies that assessed the activity of DNMT in atherosclerosis patients with hyperhomocysteinemia (95).

In addition to the passive demethylation that results from a decrease in or loss of DNMT activity, active DNA demethylation is carried out by TET enzymes (77). Upon assessment of TET activity in BMDM, our data indicated that TET activity in mice that received BM from *Pg*-inoculated donors was almost double that in mice that received BM from sham-inoculated donors. Consistent with this, the WGBS data indicated that the promotor region of *TET2* was hypomethylated, which was suggestive of overexpression.

Homocysteine, a non-protein-forming amino acid, is strategically located at the intersection of the methionine cycle and the transsulfuration pathway. The only source of homocysteine is through the methionine cycle, where methionine is converted to SAM in the presence of MAT, which is in turn demethylated to SAH by various methyltransferases (71, 76). In a reversible reaction, SAH is hydrolyzed to homocysteine. At this point, homocysteine has three options: (1) it is converted back to SAH *via* SAHH, (2) it is remethylated to methionine *via* methionine synthase, and (3) it is synthesized to cysteine through the transsulfuration pathway in the presence of the rate-limiting enzyme, cystathionine *β*-synthetase. The fate of homocysteine is governed by several factors, including diet, medications, and pathological conditions. Glutathione (GSH), the end-product of the transsulfuration pathway, is one of the fundamental antioxidants synthesized in cells (96). Hence, under conditions of oxidative stress, synthesis of cysteine and eventually GSH through the transsulfuration pathway is favored over remethylating homocysteine to methionine. Based on the WGBS data that showed hypermethylation of the genes encoding the rate-limiting enzymes MAT and SAHH, we analyzed plasma levels of SAM and SAH. SAH plasma levels were significantly higher in mice that received BM from *Pg*-inoculated donors than in those that received BM from sham-inoculated donors.

This result is expository for multiple reasons. First, it validated and strongly suggested a mechanism for our global hypomethylation data revealed by WGBS. Increased levels of SAH have been shown to directly inhibit DNMT1 activity by up to 70%, and other SAM-dependent methyl transferases by up to 90% (76). Experimentally inhibiting SAHH with adenosine-2,3-dialdehyde in endothelial cells resulted in increased intracellular SAH and decreased DNA methylation (97). Second, it suggested a mechanistic link between global hypomethylation and increased atherosclerosis. Several studies have associated plasma SAH levels with CVD and atherosclerosis (75). In a case–control study, plasma SAH levels were significantly higher in CVD patients than those in healthy controls, suggesting that plasma SAH levels could be used as a sensitive indicator for CVD risk (98). A more recent prospective cohort study on 1,003 patients undergoing coronary angiography reported a significant association between plasma SAH levels and increased risk of stroke and nonfatal myocardial infarction (99). Experimentally, in a series of studies, Luo et al. and Xiao et al. inhibited SAHH in *ApoE^o^* mice by feeding them an adenosine-2,3-dialdehyde-supplemented diet (100, 101). After 8, 16, and 24 weeks, there were significant increases in plasma SAH levels associated with the accelerated development of atherosclerotic lesions (100, 101).

Mechanistically, other than its role in passive DNA demethylation, the role of SAH in increasing atherosclerosis is not fully understood. Several studies proposed induction of oxidative stress as a potential mechanism. An *in vitro* study reported that intracellular accumulation of SAH in endothelial cells resulted in significantly higher expression of nicotinamide adenine dinucleotide phosphate oxidase and production of ROS associated with apoptosis (102). Increased plasma SAH levels in *ApoE^o^* mice, *via* methionine-supplemented diet or through inhibition of SAHH, induced proliferation and migration of vascular smooth muscle cells *via* an oxidative stress-dependent activation of the extracellular signal-regulated protein kinase pathway (100).

Our results also showed lower plasma SAM levels in mice that received BM from *Pg*-inoculated donors compared to those in mice that received BM from sham-inoculated donors. Although the difference is not statistically significant, in combination with the increased levels of SAH, the resulting SAM:SAH ratio is lower in the former mice. The ratio between SAM and SAH has been referred to as the methylation index or methionine index and has been repeatedly shown to be positively correlated with global DNA methylation levels. Hence, the lower SAM:SAH ratio in mice that received BM from *Pg*-inoculated donors was in line and possibly underlaid the global DNA hypomethylation. Importantly, this ratio has been associated with atherosclerosis and CVD (103).

To further understand the increased atherosclerosis in BM recipients of *Pg*-inoculated donors mechanistically, we focused on two apolipoproteins that were hypermethylated, *apoE* and *apoA1*. ELISA demonstrated a 34% decrease in plasma apoE in BM recipients of *Pg*-inoculated donors. ApoE regulates plasma lipid levels by binding to cellular lipoprotein receptors, including the LDLR, VLDL receptor, and the LDL receptor-related protein (79). In *LDLR^o^* mice, a decrease in apoE binding to these receptors exacerbates LDL cholesterol accumulation in plasma, as confirmed by lipoprotein profiling (an 89% increase in VLDL cholesterol and a 20% increase in LDL cholesterol). ApoE is also important in preventing oxidation, including lipid oxidation of LDL to oxLDL, which is proatherogenic (104). Lipoprotein analysis also showed a 28% decrease in HDL cholesterol in BM recipients of *Pg*-inoculated donors. HDL has both antioxidant properties and protects against atherosclerosis through reverse cholesterol transport (105). The changes observed were in line with the increased atherosclerotic lesion burden.

Based on our results, we propose that *Pg*-induced PD in the atherosclerotic *LDLR^o^* mouse model, possibly through oxidative stress, which has been shown to increase in PD patients, creates epigenetic pressure in BM progenitor cells (106–108). This epigenetic pressure manifests as global DNA hypomethylation and is associated with increased atherosclerosis. One potential mechanism is through SAH-driven disruption of the methionine cycle. Whether oxidative stress, either *Pg*-induced or as a result of increased SAH, underlies the initiation and sustainability of this disruption, as well as the role of SAHH, are yet to be examined.

## Materials and methods

### *Pg* infection, high-fat diet, BM transplantation, and assessment of chimerism

All chemicals were from Fisher Scientific unless otherwise specified. All animal procedures were prior approved by the University of Alberta Animal Care and Use Committee (Protocol #0570). *LDLR^o^* mice were commercially obtained, bred, and housed under specific pathogen-free conditions (B6.129S7-*LDLR^tm1Her^*/J, The Jackson Laboratory, IMSR JAX:002207, RRID:IMSR_JAX:002207). *Pg* bacteria (33277, American Type Culture Collection) were grown to saturation (10^9^ cells/mL) at 37°C in Schaedler's broth containing vitamin K and hemin (L007496, BBL) under anaerobic conditions using AnaeroPack-Anaero (R681001, Mitsubishi Gas Company). Eight-week-old mice of both sexes were anesthetized with ketamine [100 mg/kg intraperitoneally (IP)] and xylazine (10 mg/kg IP). The bacteria were concentrated to 10^10^ cells/mL in a phosphate-buffered saline (PBS) solution containing 2% carboxymethylcellulose (a thickener used in the food industry to promote tooth adherence). Two hundred microliters of this was applied to the gums and molars*.* Coincident with the first inoculation, mice were fed a high-fat diet (21% fat, 0.15% cholesterol; 88317, Teklad, Envigo) to promote atherosclerosis, which continued for 16 weeks. Control mice were sham-inoculated and fed a high-fat diet for 16 weeks. The number of recipient mice/group was determined by a power calculation based on a pilot experiment in male mice. The sample size was calculated to be 9/group (80% power, alpha = 0.05). Extra mice were included in case of dermatitis/malocclusion.

*LDLR^o^* mice of both sexes (8 weeks of age) were irradiated (lethal dose) following a standard protocol (2 × 5.5 Gray, Gammacell 1000 Elite irradiator) used successfully by our lab and others (109). Irradiated mice received ∼1 × 10^7^ sex-matched BM cells by retro-orbital injection from mice inoculated with *Pg* and fed a high-fat diet or from mice sham-inoculated and fed a high-fat diet. After transplantation, recipient mice were fed a normal chow diet for 4 weeks to allow for complete engraftment and turnover of hematopoietic cells and then the high-fat diet for 16 weeks.

We used PCR of DNA extracted from white blood cells to assess chimerism following BM transplantation in the control experiment, where *CD36^o^* mice were transplanted with wild-type BM. The primers for the wild-type allele are EG1 5′ CAG CTC ATA CAT TGC TGT TTA TGC ATG 3′ and EG2 5′ GGT ACA ATC ACA GTG TTT TCT ACG TGG 3′; the product is ∼600 base pairs. The primers for the knockout allele are the EG1 primer above and EG3 5′ CCG CTT CCT CGT GCT TTA CGG TAT C 3′; the product is ∼800 base pairs.

In a complementary experiment, wild-type mice were transplanted with *CD36^o^* BM. Four weeks later, whole blood, obtained by cardiac puncture, was treated twice with erythrocyte lysis buffer. Washed pellets were resuspended in PBS containing 2 mM ethylenediamine tetraacetic acid disodium salt (EDTA) and 0.5% bovine serum albumin. Fluorophore-conjugated antibodies with specificity to mouse antigens are as follows: anti-CD11b (M1/70, 550993, BD Biosciences, RRID:AB_394002), anti-CD11c (HL3, 561022, BD Biosciences, RRID:AB_2033997), and anti-CD36 (JC63.1, 10009870, Cayman Chemical, RRID:AB_10342682). Dead cells were excluded using live/dead fixable cell stains. The CD11b+/c + fraction was analyzed for CD36 expression. Paraformaldehyde-fixed cells were acquired using a Becton Dickinson LSR Fortessa flow cytometer (University of Alberta Faculty of Medicine and Dentistry Flow Cytometry Core Facility, RRID:SCR_019195) and analyzed with FlowJo (version 10) software (RRID:SCR_008520).

### Aorta morphometry

Mice were euthanized by pentobarbital overdose (200 mg/kg, IP), perfused first with 10 mL of PBS and then 5 mL of buffered formalin (Formalde-Fresh). The complete aorta, including the subclavian and left and right carotid arteries, was dissected free of fat from the mouse and then postfixed in Formalde-Fresh at 4°C. Aortae were transferred to PBS after 24 h and stored at 4°C. Neutral lipids in aortic plaque were stained with oil red O (00625, Millipore Sigma) by incubation at room temperature for 15 min, followed by 1–2 min destaining in methanol. Each aorta was placed open on a microscope slide, covered with a coverslip, hydrated with PBS, and digitally scanned using a CanonScan LiDE 210 scanner at 1,200 dpi and a depth of 24 bits. Three independent measures of lesion area (red pixels) were chosen and averaged for each aorta in a blinded fashion using Adobe Photoshop software (RRID:SCR_014199). Likewise, the total aorta area (red + white) was chosen. The lesion area was represented as the mean percentage of the total aortic area.

A subset of hearts was cryostat sectioned (5 μM), and serial sections were stained either with oil red O or Masson's trichrome. Similarly sized lesions (*n* = 5/group) were selected for further analysis. Relative amounts of collagen (blue staining) or foam cell (red staining) areas were assessed using Adobe Photoshop software (RRID:SCR_014199) and expressed as a percentage of total lesions.

### Macrophage isolation, blood collection, qPCR, and cytokine profiling

Elicited macrophages were isolated by peritoneal lavage into sterile PBS 4 days following intraperitoneal injection of 2 mL of 4% sterile Brewer's thioglycolate (Difco). More than 90% of cells are macrophages at this time point (110). After centrifugation, cells were frozen at −20°C. Blood was collected into EDTA (1.6 mM final concentration) from the heart; plasma was stored at −20°C for future analyses.

Reagents for qPCR were purchased from Bio-Rad: PrimePCR PreAmp for the SYBR Green assay, PrimePCR Template for the SYBR Green Assay, and primer pairs for the genes IL1beta (qMmuCED0045755), IL6 (qMmuCED0045760), TNFalpha (qMmuCED0004141), and GAPDH (qMmuCED0027497). Each reaction consisted of 1 μL of 20× PrimePCR assay, 10 μL of 2× SsoAdvanced Universal SYBR Green Supermix (1725271, Bio-Rad), 4 μL of cDNA sample and 5 μL of nuclease-free water. A positive control using the respective template and a negative control were included. Reactions were run on a Bio-Rad C1000 touch thermal cycler (Bio-Rad CFX96 real-time PCR detection system, RRID:SCR_018064) using the following protocol: step 1: 95°C × 1 min; step 2: 95°C × 5 s; step 3: 60°C × 30 s. Repeat steps 2 and 3 for 40 cycles. The melt curve was completed from 65°C to 95°C (0.5 increments), 5 s/increment. Data were analyzed using CFX Manager software (RRID:SCR_017251).

To determine the ideal housekeeping gene, we used reference gene M96 (Bio-Rad), a predesigned 96-well panel preplated with 14 commonly used housekeeping genes. Glyceraldehyde 3-phosphate dehydrogenase (GAPDH) was selected as our housekeeping gene based on stability in our experimental conditions.

The relative expression levels of 40 mouse cytokines were assessed using the Mouse Cytokine Array Panel A (R&D Systems, ARY006; five pooled samples/group), following the manufacturer's instructions. A Bio-Rad ChemiDoc MP Imaging System (RRID:SCR_019037) and Image Lab 5.0 software (Bio-Rad, RRID:SCR_014210) were used to capture and analyze the images. The average density of the duplicate spots representing each cytokine was determined, then, the density of the negative control spot was subtracted from that value.

### Isolation of DNA, RNA and complementary DNA synthesis

For DNA isolation, the macrophage pellet from each individual mouse was incubated in 200 μl of 10 mM Tris, pH 7.5, 300 mM sodium chloride, 5 mM EDTA, and 1% sodium dodecyl sulfate containing 100 μg/mL proteinase K at 55°C overnight and then vortexed. Two hundred microliters of tris-saturated phenol were then added to the lysate and vortexed, followed by centrifugation for 5 min at 22,000 × *g.* The aqueous layer (top) was then removed to a new tube, and 200 microliters of chloroform was added, vortexed, and then centrifuged as previously. The aqueous layer (top) was removed to a new tube, and 2 volumes of 100% ethanol were slowly added. DNA was collected by centrifugation as before. The supernatant was removed, and the DNA pellet was air-dried and then resuspended in 10 mM Tris, pH 8 and 1 mM EDTA + 2 μg/mL RNase A. The purity and concentration were assessed by Thermo Fisher Qubit 2.0 Fluorometer Qubit 2.0 analysis (RRID:SCR_020553) and agarose gel electrophoresis.

RNA was purified using the RNeasy Mini Kit (74104, Qiagen) following the manufacturer's protocol. The purity and concentration were assessed as above. Complementary DNA was synthesized from purified RNA using the RT^2^ First Strand Kit (330404, Qiagen) following the manufacturer's protocol.

### Whole genome bisulfite sequencing

Novogene Co. Ltd. performed all sequencing, library construction, analysis, and quality control. We pooled equal quantities of male macrophage DNA from five donors/group. Bismark software (RRID:SCR_005604) was used to align bisulfite-treated reads to the mouse reference genome (63).

### Generation of BM-derived macrophages and preparation of L929 conditioned media

Mice were euthanized with pentobarbital (200 mg/kg IP). The femur and tibia were removed, cleaned, and flushed with Iscove's modified Dulbecco's media (Cat# 12440053, Gibco) containing 10% fetal bovine serum (FBS). The BM cells were then centrifuged (5 min @ 3,800 × *g*), and the pellet was resuspended at a concentration of ∼10^7^ cells per cryovial in a mixture of 90% FBS and 10% dimethyl sulfoxide (DMSO). Cells were stored at −80°C.

BMDM were generated following previously published protocols (111). To dilute out the DMSO, frozen BM cells were resuscitated by gently thawing at 37°C and then transferred to a 15-mL tube. Drops of resuscitation media (Roswell Park Memorial Institute (RPMI) media (SH 30255.01, Hyclone) containing 100 units/mL penicillin, 100 μg/mL streptomycin, 2 mM L-glutamine, and 10% FBS) to 9 mL were dropped along the tube wall. Cells were centrifuged for 5 min at 870 × *g* and resuspended in 10 mL of resuscitation media. Cells were plated in 100 mm bacterial plates (2 plates/sample) to allow for easier detachment and incubated at 37°C in 5% CO_2_. On the fourth day following the initial culture, resuscitation media was removed and 10 mL of differentiation medium [resuscitation media containing 15% L929 conditioned media (LCCM)] were added to the culture, and macrophages were allowed to develop.

To generate LCCM, cells were purchased from ATCC (NCTC clone 929) and cultured according to their protocol. Briefly, cells were resuscitated and expanded to near confluency in Dulbecco's modified Eagle's medium (DMEM) (SH 30081.01, Hyclone) containing 100 units/mL penicillin, 100 μg/mL streptomycin, 2 mM L-glutamine, and 10% FBS. At day 10, the amount of media was doubled, and the cells were cultured for an additional 10 days. On day 20, media was collected, sterilized through a 0.2 μm filter, aliquoted, and stored at −20°C.

### Nuclear extract preparation, DNMT and TET activity assays

Nuclear extracts of BMDM were prepared using the EpiQuik Nuclear Extraction Kit (OP-0002-1, Epigentek), following their protocol, generating nuclear and cytoplasmic extracts. The protein concentration of the nuclear extract was determined using the bicinchoninic acid protein assay (PI23235, ThermoFisher Pierce), following the manufacturer's protocol. To determine DNMT activity, we used the EpiQuik DNA Methyltransferase Activity/Inhibition Assay Kit (*P*-3001-2, Epigentek) and followed the manufacturer's protocol. BMDM nuclear extracts were used in this assay. To determine TET activity, we used the Epigenase 5mC-Hydroxylase TET Activity/Inhibition Assay Kit (*P*-3086-96, Epigentek) and followed the manufacturer's protocol. BMDM nuclear extracts were used in this assay.

### S-adenosylmethionine and S-adenosylhomocysteine plasma levels

We measured the plasma levels of S-adenosylmethionine (SAM) and S-adenosylhomocysteine (SAH) using an ELISA Combo Kit (MET-5151-C, Cell Biolabs), following the manufacturer's instructions. At the time of sacrifice, plasma from 3 mice/group was collected. Using Magne Protein A Beads (G8781, Promega), IgG was removed from plasma samples prior to measuring SAM and SAH levels as per the manufacturer's instructions. DNMT activity, TET activity, and SAM and SAH experiments were performed on a different mice cohort from that used in the atherosclerosis study. BM cells and plasma were collected ∼11 months after BM transplantation.

### ApoE ELISA and lipoprotein profiling

ApoE was detected in plasma using the Apolipoprotein E SimpleStep ELISA Kit (ab2155086, Abcam). For lipoprotein analysis, fresh plasma was provided to the University of Alberta Faculty of Medicine and Dentistry Lipodomics Core (RRID:SCR_019176).

### Statistical analysis

The differences in donors' gene expression and atherosclerosis lesion burden, DNMT activity, TET activity, and SAM and SAH plasma levels between the groups were statistically compared using an unpaired *t*-test if samples passed the D'Agostino and Pearson omnibus normality test or a Mann–Whitney *U* test if they were not normally distributed, with significance set at *p* < 0.05. GraphPad Prism software (RRID:SCR_002798) was used for all statistical analyses.

## Data Availability

The datasets analyzed for this study can be found in the NCBI-GEO repository, accession #GSE229568.
