# Supplementary material for: DNA methylation changes underlie the long-term association between periodontitis and atherosclerotic cardiovascular disease
Source: Front Cardiovasc Med. 2023 Apr 21;10:1164499. doi: 10.3389/fcvm.2023.1164499 (PMC10160482; doi:10.3389/fcvm.2023.1164499)
Supplement: Supplementary file 5 [file Datasheet1.pdf]

Supplementary Material

DNA Methylation Changes Underlie the Long Term Association Between Periodontitis and Atherosclerotic Cardiovascular Disease

Mohamed Omar, Maria Alexiou, Umar R. Rekhi, Konrad Lehmann, Aneesh Bhardwaj, Cole Delyea, Shokrollah Elahi, Maria Febbraio*

*** Correspondence:** Maria Febbraio: febbraio@ualberta.ca

Supplementary Figures

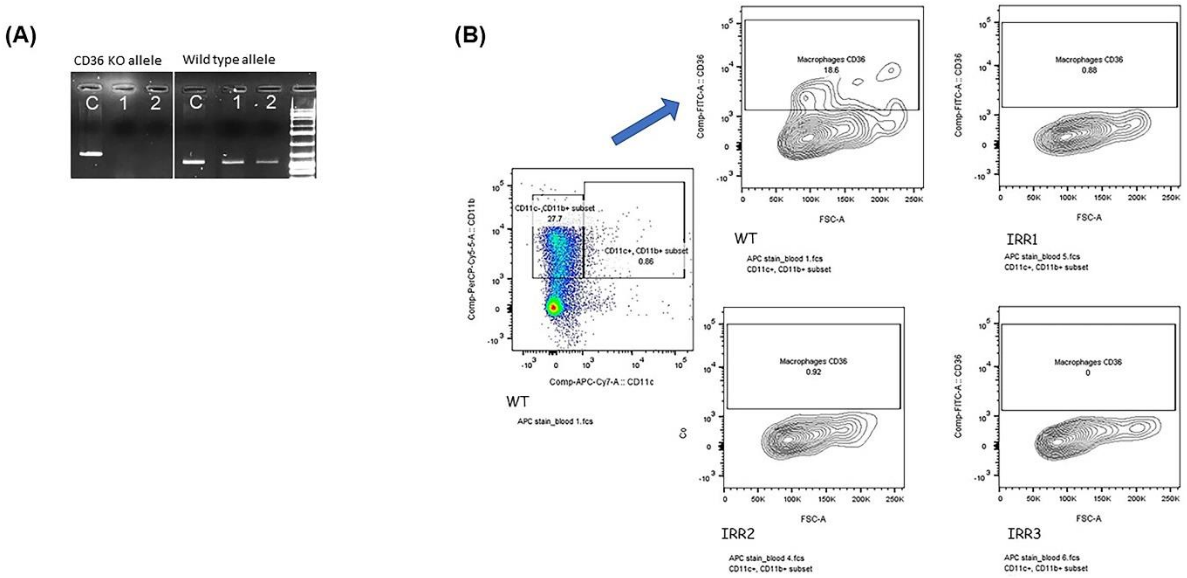

Supplementary Figure 1. (A) Assessment of chimerism by PCR/agarose gel electrophoresis. *CD36^{o}* mice (n=2) were lethally irradiated and transplanted with BM from wild type mice. 4 weeks later, DNA from white blood cells was isolated for PCR. Under conditions where control (C) DNA amplified, only a band for the wild type allele amplified in BM recipient mice (1, 2). **(B)** Assessment of chimerism on macrophages by flow cytometry. Wild type mice were lethally irradiated (IRR) and transplanted with BM from *CD36^{o}* mice (IRR1, IRR2, IRR3). 4 weeks later blood was collected and gated for macrophages using antibodies for CD11b and CD11c. The gating strategy is shown in the first panel. In wild type mice (WT), 18.6% of the CD11b+, CD11c+ cells expressed CD36. Less than 1% of cells from transplanted mice showed CD36 expression.

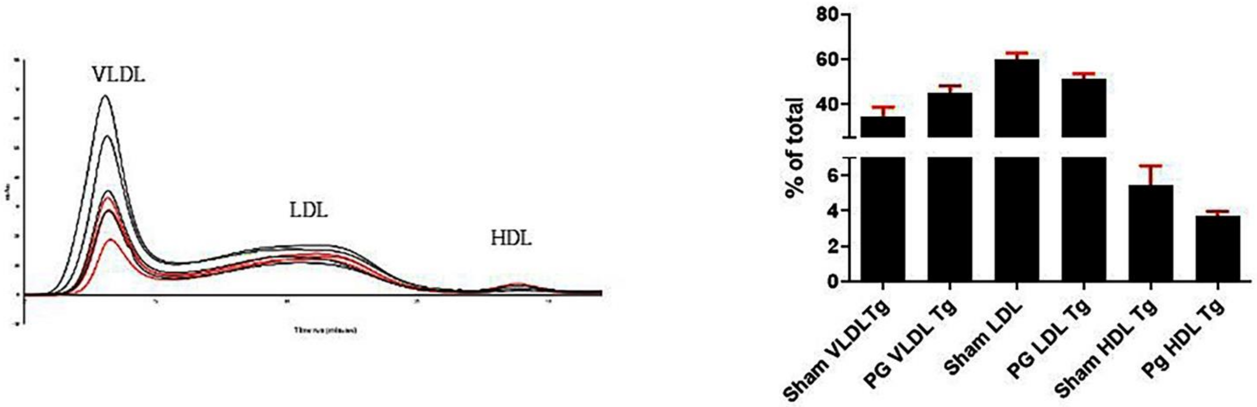

Supplementary Figure 2. Chromatographs of FPLC separation of lipoproteins and triacylglycerol analysis from male mouse recipients of Sham or Pg BM (n=3 Sham; n=4 Pg). Quantification of peaks as percent of total is shown in adjacent bar graph.
